# Hidradenitis Suppurativa (HS) prevalence, demographics and management pathways in Australia: A population-based cross-sectional study

**DOI:** 10.1371/journal.pone.0200683

**Published:** 2018-07-24

**Authors:** Miriam Calao, Jodie L. Wilson, Lynda Spelman, Laurent Billot, Diana Rubel, Alan D. Watts, Gregor B. E. Jemec

**Affiliations:** 1 Abbvie Pty Ltd, Mascot, New South Wales, Australia; 2 Veracity Clinical Research, Woolloongabba, Queensland, Australia; 3 Sydney Medical School, University of Sydney, Sydney, New South Wales, Australia; 4 The George Institute for Global Health, Sydney, New South Wales, Australia; 5 Woden Dermatology, Canberra, Australian Capital Territory, Australia; 6 Department of Dermatology, Roskilde Hospital, Health Sciences Faculty, University of Copenhagen, Roskilde, Denmark; University of Naples, ITALY

## Abstract

**Background:**

Hidradenitis Suppurativa (HS) is a painful, chronic inflammatory skin disease. Global estimates of prevalence vary between 0.03% and 4% of the population.

Our main aim was to determine HS prevalence in the Australian adult population focussing on the demographics, management pathways and diagnosis rate of individuals living with HS.

**Methods:**

In this population-based cross-sectional study, 17,050 individuals representative of the Australian adult population were asked through face-to-face household interviews to answer a previously validated HS screening questionnaire with high diagnostic power. Individuals who screened positive were asked additional questions, including previous diagnosis of HS and number/type of physicians consulted regarding their condition.

**Results:**

11,433 Australian residents answered the HS questionnaire, 88 screening positive for HS (0.77%; 95% CI 0.62–0.95). Considering the previously reported sensitivity (0.97) and positive predictive value (0.85) of the screening questionnaire, HS prevalence was estimated to be 0.67% (95% CI 0.53%-0.84%). 6 of 88 suspected HS individuals reported a pre-existing HS diagnosis (6.8%; 95% CI 3.2%-14.1%). 25.6% of the undiagnosed individuals suspected of having HS had not seen any clinicians regarding their boils; the remaining ones had consulted General Practitioners (96.7%), and clinicians from different specialties. Comparisons of individuals who screened positive for HS versus those who screened negative demonstrated statistically significant differences in gender (p = 0.0046), age (p<0.0001), BMI (p = 0.0307), smoking status (p<0.0001), employment status (p<0.0001) and income (p = 0.0321).

**Conclusions:**

The prevalence of HS in Australia was estimated to be 0.67% (95% CI 0.53%-0.84%). The diagnosis rate amongst the suspected HS cases was low, which appeared to be due to a combination of patients not seeking help and decentralization of care. Individuals suspected of having HS were more likely to be females, young, obese, smokers, unemployed or at home duties and having lower annual personal income in comparison with individuals not suspected of having HS.

## Introduction

Hidradenitis Suppurativa (HS) (also known in the past as *acne inversa*) is a chronic, progressive, inflammatory and relapsing skin disease with a severe negative impact on Quality of Life (QoL) and often associated with low socioeconomic status [1, 2]. It is characterized by painful inflammatory lesions mainly located in the inverse body areas, most commonly the axillae, groin, buttocks, and inframammary areas [3]. The lesions consist of nodules which can progress to abscesses, sinus tracts (tunnels) and scarring [4].

The disease causes obvious morbidity and its severity is usually classified using the Hurley staging according to the most severely affected region [5]:

- Stage I (Mild)—Abscess formation, single or multiple, without sinus tracts and cicatrization
- Stage II (Moderate)—Recurrent abscesses with tract formation and cicatrization, single or multiple, and widely separated lesions
- Stage III (Severe)—Diffuse or near-diffuse involvement or multiple interconnected tracts and abscesses across the entire area

Currently, HS is often managed and/or diagnosed by many different specialties, such as surgeons, emergency physicians, plastic surgeons, infectious disease specialists, general practitioners, and dermatologists [6]. Nevertheless, the mean time between symptom onset and HS diagnosis has been reported to be 7.2 years in a wide range of countries [7], and HS patients are reportedly often misdiagnosed or undiagnosed [8]. This may indicate that not all clinicians are familiar with the disease and/or that many patients do not consistently seek medical help [1]. Because of the severe impact that HS has on patients, it is particularly important to understand prevalence and diagnosis rates of HS, as well as the profile and characteristics of people living with HS and their management pathways. This knowledge could assist in developing strategies to inform physicians and patients of the disease and identify patients affected by it, with the ultimate aspiration of preventing progression, disfigurement, pain, and disability.

Several studies have attempted to determine HS prevalence in different settings and using different methods. The result is a set of highly variable estimations of HS prevalence, ranging between 0.03% and 4% of the population [6, 9–11]. Three types of studies have been pursued so far: (I) registry-based studies, (II) prospective examination of patients, and (III) self-reported data from larger groups (e.g. population-based studies) [6]. These different designs each have their strengths and weaknesses. However, the most reliable prevalence estimates come from cross-sectional, general population-based studies using validated screening questions and/or clinical assessment. Patients most often describe the disease as ‘‘boils” [12], and it has been proposed that the clear symptomatology (painful lesions recognized by patients as boils), the specific areas affected, and the recurrence/chronicity of the lesions are sufficient for reliable, self-reported diagnosis [13, 14]. Three different questionnaires based on those criteria have been tested and validated by Esmann and colleagues [13] and shown to have high diagnostic power to identify individuals affected by HS and can therefore be used as a tool for population-based epidemiology studies of HS.

There is also uncertainty around the estimations of HS severity distribution. Hurley stage I and II disease have been reported to be the most common among diagnosed HS individuals, affecting between 24–68% and 28%-54% of HS patients respectively, depending on the study [10, 15–19]. Stage III is less common, occurring in 2–29% of HS patients [10, 15–19]. These studies are however subject to selection bias as they mainly reflect the severity distribution among HS patients under care and therefore have not taken into account the undiagnosed pool of HS patients. The development and validation of a self-administered questionnaire able to predict the severity of HS would offer advantages over physical assessment in population-based epidemiologic research and allow for the estimation of HS severity distribution amongst the wider HS population.

Here we aimed to determine HS prevalence in the Australian adult population through a population-based cross-sectional study using a previously validated HS screening questionnaire with high diagnostic power [13]. HS diagnosis rate as well as demographics, profile and management pathways of individuals living with HS were also assessed. In parallel, with the aim of enabling the estimation of HS severity distribution in our study population, we also performed a cross-sectional observational study to assess the ability of a newly developed self-administered HS severity questionnaire to predict HS severity as defined by the Hurley staging system.

## Methods

The project reported here consisted of two separate studies: (I) A cross-sectional observational study to assess the ability of an experimental, newly developed, self-reported HS severity questionnaire to predict Hurley Stage in HS patients; and (II) a multi-stage cross-sectional observational study to determine the prevalence of HS in the Australian population.

### Design

#### Validation of an experimental self-administered questionnaire to predict the severity of HS: A cross-sectional observational study

An experimental self-administered HS severity questionnaire (Table 1) was developed by the authors based on physical and social characteristics of HS that were believed to be able to distinguish between the 3 Hurley stages of the disease. A broad spectrum of questions were devised covering all anticipated disease features that could be readily reported by patients with the objective of narrowing the list of questions post-study. A total of 117 consecutive patients presenting to 10 Australian dermatology clinics with a diagnosis of HS and age of 18 or older were enrolled in this study. Approximately one-third of HS patients were recruited for each Hurley stage (N = 39 Stage I, N = 43 Stage II, and N = 35 Stage III). During a single study visit, the study subjects completed both an experimental, newly developed self-administered HS severity questionnaire (Table 1) and DLQI (Dermatology Life Quality Index), and underwent a physical assessment by their treating dermatologist for assessing the severity of the disease based on Hurley staging. Demographics and medical history were also collected for each patient: concomitant medical conditions, duration of HS symptoms, time since diagnosis and medications used to treat HS were recorded in a short form.

**Table 1:** Experimental HS severity questionnaire.

**1.**	**In the last 6 months, how many sore or painful boils/lumps at least 1 cm (or half an inch) in diameter have you had?**											
	a. 0	b. 1	c. 2–3	d. 4–6	e. >6						
**2.**	**In the last 6 months, what is the most pain you have experienced from your boils or lumps *(0 is no pain and 10 is unbearable pain)*?**											
	NO PAIN								WORST PAIN I CAN IMAGINE		
	☐ 0		☐ 1	☐ 2	☐ 3	☐ 4	☐ 5	☐ 6	☐ 7	☐ 8	☐ 9	☐ 10
**3.**	**Have you ever had boils or lumps heal leaving scars that feel harder than the skin around the scars? (at any stage in your life, not just the last 6 months)**											
	☐ Yes		☐ No									
**4.**	**In the last 6 months, have the boils or scars:** ***(tick Yes or No for each question)***											
	a. Restricted your movements? ☐ Yes ☐ No											
	b. Interfered with your work/school activities? ☐ Yes ☐ No											
	c. Caused you embarrassment or shame? ☐ Yes ☐ No											
	d. Impacted on personal or physical relationships? ☐ Yes ☐ No											
**5.**	**Does the skin of the area affected ever heal completely possibly leaving scars)?**											
	☐ Yes		☐ No									
**6.**	**Does the area affected by the scarring ever feel “lumpy” or the contour of “bubble wrap”?**											
	☐ Yes		☐ No									
**7.**	**How big is the area currently affected by scarring that you would regard as ugly/distressing?**											
	a. Surface area of 3 hands or larger.											
	b. Surface area of less than 3 hands, but more than 2 hands.											
	c. Surface area of 2 hands or less.											
	[Note. You are being asked to use your hands as tools to measure the size of the affected area on other parts of your body. Place your hand (including fingers) over the affected area to estimate the surface area, and repeat until the affected area/s have been covered.]											
**8.**	**Does the area currently affected always contain some painful pus-filled boils, lumps and scars?**											
	☐ Yes		☐ No									

#### HS Epidemiology in Australia: A population-based cross-sectional observational study

**Fig 1:**
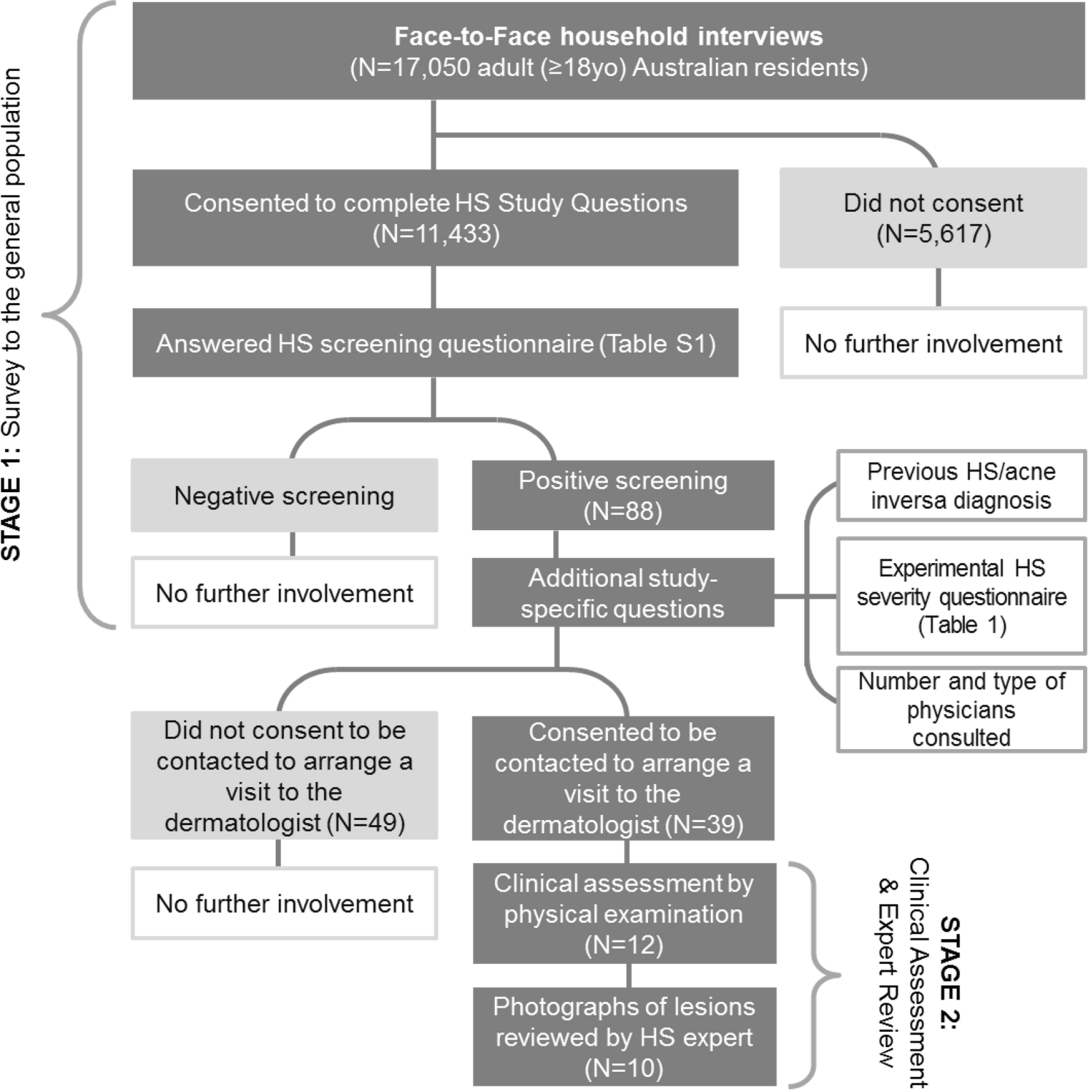
HS Epidemiology study flow chart.

This study consisted of two stages: Stage 1—Survey of the adult general population; Stage 2 –Clinical assessment and Expert Review (Fig 1).

In Stage 1 of the epidemiology study, all adult (≥ 18yo) Australian residents participating in the Single Source establishment survey run by Roy Morgan Research Ltd [20] were eligible to be included. The single source sample is representative of the Australian population aged 14 years and older in terms of gender, age and geographic location. The sample is constructed using random sample and geographical stratification to cover all states and territories. In addition to the standard questions administered in the Roy Morgan Single Source Establishment Survey, which include demographic characteristics, study specific questions were administered to those who consented. The survey was conducted face-to-face, and a total of 17,050 individuals interviewed between the 1^st^ of August 2015 and the 11^th^ of December 2015 were asked for consent to be administered the study questions. The survey provider Roy Morgan is governed by professional standards set out in the Code of Professional Behaviour of the Australian Market and Social Research Society and ethical standards as set out by Ethical Standard Opinions and Marketing Research Professionals.

Consenting individuals firstly responded to the HS screening questionnaire previously validated by Esmann et al (“Do you repeatedly have outbreaks of big sore or painful nodules or boils that heal with scars in any of these locations: Groin, Armpits, Sexual organs, Anal region, Under the breasts, Folds on the Stomach/around the navel”), which has high sensitivity and positive predictive value (SE 0.97; PPV 0.85) [13]. As shown in S1 Table (Supporting Information), the questionnaire used in this study, as opposed to the original version from Esmann and colleagues, also listed “Other locations”. However, in line with the validated questionnaire, a suspected HS subject had been defined as an individual with presence of outbreaks AND at least one nodule/boil location other than “Other locations”. In our survey, the text was also supplemented by a visual diagram showing the characteristic locations of boils/nodules listed in the questionnaire in order to overcome any language barriers (S1 Table, Supporting Information).

Individuals suspected of having HS based on the HS screening questionnaire, also completed the experimental HS severity questionnaire (Table 1) and were asked a different set of additional study questions depending on whether they reported having been previously diagnosed with HS/*acne inversa* (S2 Table, Supporting Information). These questions included number and type of physician(s) consulted regarding the condition of boils.

In Stage 2 of the epidemiology study, all interviewed subjects who screened positive for the HS screening questionnaire were invited to attend a visit with a dermatologist for clinical assessment. Consenting subjects were contacted to schedule a visit at the closest dermatology clinic among the 8 sites participating in the study (1 site in the Australian Capital Territory, 2 sites in the state of New South Wales, 1 site in the state of Queensland, 1 site in the state of Western Australia, 2 sites in the state of Victoria, and 1 site in the state of South Australia).

Individuals attending the dermatology clinic underwent physical examination by a dermatologist between November 2015 and February 2016 to confirm HS diagnosis, assess the severity of the disease, and collect additional study-related information such as duration of symptoms, presence of comorbidities, abscess and inflammatory nodule count (AN count), past and current treatments for HS.

De-identified photographs of the lesions were uploaded onto a secure server for blinded review by an international independent HS expert, with the goal of confirming both HS diagnosis and Hurley Staging.

### Ethics and informed consents

#### HS severity questionnaire validation study

Ethics approval for the study was obtained through the following HREC committees: Melbourne Health, Westmead Hospital, Bellberry limited and St. Vincent’s Hospital Melbourne. Study participants received a unique study identification number, issued at the time informed written consent was obtained.

#### HS Epidemiology study

The study protocol, any amendments, the informed consents and other information that required pre-approval were reviewed and approved by a central ethics committee (Bellberry Limited). Informed consent of the participants was required. Informed consent was obtained from the subjects (i) to administer the study-specific questions during the household Roy Morgan survey in Stage 1 of the study (ii) where responses indicated a possible HS diagnosis, to obtain contact details to schedule a visit at the closest dermatology clinic (iii) at the dermatology clinic, prior to the clinical assessment. All consents were written and signed by the study subjects except for the household survey, where subjects’ consent or dissent to answer the HS questions was recorded by the Roy Morgan staff into a Computer Assisted Personal Interviewing (CAPI) device, which was used to record the other answers to the Roy Morgan survey questions. Only data from consenting subjects was received. Data collected during the prevalence survey remained de-identified by the survey provider for participants screened as not having HS. Participants screening positive for HS and who agreed to attend a clinical consult, were issued a unique participant identification number.

### Outcome measures

#### HS severity questionnaire validation study

The study outcome measures were Hurley Stage, DLQI and the completed experimental HS severity questionnaire. The DLQI and the experimental HS severity questionnaire were self-administered; Hurley Stage was determined via physical assessment.

#### HS Epidemiology study

The primary objective of the study was to calculate the crude HS prevalence, estimated using the number of participants who screened positive for HS during Stage 1 (Population Survey). The secondary objectives were to calculate the HS severity distribution; and the combined prevalence of moderate and severe HS as determined by the experimental HS severity questionnaire administered in Stage 1 subject to its validation. The exploratory outcome measures were: number of participants diagnosed with HS during Stage 2 (Clinical Assessment & Expert Review); the HS severity distribution as determined by the clinician in Stage 2; pathways to accessing clinical care; history of previous treatment; diagnosis rate among people living with HS; other health problems, duration of the condition and treatments used, per severity level; number of participants diagnosed with HS during the expert review of photographs; and the HS severity distribution as determined by the expert reviewer.

### Statistical methods

#### HS severity questionnaire validation study

A sample size of 40 patients per stratum (Hurley Stage I, II and III) was calculated to be able to estimate sensitivity/specificity parameters with a precision ranging between 0.13 and 0.19 if the sensitivity and specificity parameters were equal to 0.9. The sensitivity and specificity of the self-administered questionnaire and DLQI to predict the severity of HS were assessed against the Hurley Stage.

The utility of each of the 8 questions included in the questionnaire was evaluated by fitting each question in a univariate multinomial logistic regression model of Hurley Stage (mild, moderate, or severe) and examining the association between each individual question and Hurley Stage. The 8 questions were also included in a multivariate multinomial logistic regression model to determine if any significant associations in the univariate setting remained significant after adjusting for the other questions. This approach was repeated using logistic regression (for Hurley Stages “mild” versus “moderate or severe”; and “mild or moderate” versus “severe”).

After examining the association between the questions and Hurley Stage, the questionnaire was used to predict Hurley Stage. The 8 questions of the questionnaire were included in a multivariate multinomial logistic regression model to give a predicted probability of the 3 Hurley stages for each patient. The Hurley stage with the highest predicted probability was selected as the predicted category for each patient. The predictive ability of the multinomial logistic regression model to correctly classify the patients into the 3 Hurley stages (mild, moderate, or severe) was assessed using the Kappa coefficient. The predictive ability of the model to classify the patients into 2 Hurley stages (“mild” versus “moderate or severe”; and “mild or moderate” versus “severe”) was assessed using sensitivity and specificity. This approach was repeated using logistic regression (for 2 Hurley stages only) and discriminant analysis (for both 3 and 2 Hurley stages).

ROC curves were plotted for the multinomial logistic regression model, and logistic regression models to determine the predictive ability of the models at different decision thresholds. For example, in considering 2 Hurley stages, a patient was classified into the category with the highest predicted probability. This is equivalent to using a decision threshold of 0.5. The ROC curves plot the results for 100 thresholds from 0.01 to 100.

The overall DLQI score was also used to predict Hurley stage, where patients with a total DLQI score of 0–5 were predicted to be mild, 6–10 were predicted to be moderate, and 11–30 were predicted to be severe. This categorization was consistent with the results of the average DLQI scores of HS patients in a previous Canadian study of 2.8 (SD = 2.4) for patients with Hurley stage of mild, 8.3 (SD = 7.9) for patients with Hurley stage moderate, and 17.6 (SD = 8.0) for patients with Hurley Stage severe [21]. In a similar approach to the questionnaire, the ability of the DLQI to predict Hurley stage, was assessed using the Kappa coefficient. The predictive ability of DLQI to classify the patients into 2 Hurley stages (“mild” versus “moderate or severe”; and “mild or moderate” versus “severe”) was assessed using sensitivity and specificity.

ROC curves were plotted to determine if the decision thresholds for DLQI score based on the Canadian study were a reasonable approach, compared to using other decision thresholds.

#### HS Epidemiology study

Sample size calculation: Based on an anticipated prevalence of 1%, a sample size of 12,000 provided an exact 95% confidence interval with an absolute width of 0.36% (i.e. 0. 83% to 1.19%). For the distribution of severity, assuming a split of 40% vs 40% vs 20% for mild, moderate and severe cases respectively, 95% confidence intervals were expected to have a width comprised between 0.17% and 0.23%. Furthermore, 12,000 participants provided a 99% chance of getting at least 95 HS cases overall and at least 13 severe cases. With an assumption of 10% severe cases, 12,000 participants provided a 99% chance of getting at least 4 severe cases and a 90% probability of getting 8 or more severe cases.

The prevalence of HS was calculated as the number of subjects who screened positive for suspected HS according to the screening questionnaire (S1 Table). These subjects reported repeated outbreaks of big sore or painful nodules or boils that heal with scars in at least one of these 6 locations: groin, armpits, sexual organs, anal region, under the breasts, folds on the stomach ⁄around the navel. A 95% confidence interval for the prevalence estimate was obtained using the binomial Wilson method. Continuous variables were described for the number of participants with valid observations and using mean, standard deviation (SD), median, and quartiles, minimum and maximum. Categorical variables were described using frequencies, denominators and percentage per class level. Socio-demographic characteristics were compared between those with suspected HS and those without using chi-square tests for categorical variables and t-tests for continuous variables. A post-hoc analysis comparing the prevalence and boils locations between men and women was performed using a chi-square test.

## Results

### I. Validation of an experimental self-administered questionnaire to predict the severity of HS

The ability of an experimental HS severity questionnaire (Table 1) and DLQI to predict Hurley Stage was assessed in a cross-sectional observational study enrolling 117 individuals affected by HS attending dermatology clinics in Australia.

#### Patients’ characteristics

Approximately one-third of HS patients were recruited for each Hurley stage. Patient demographics and DLQI scores by Hurley Stage are presented in Table 2.

**Table 2:** Demographics, duration of the disease and DLQI scores by Hurley Stage of HS patients enrolled in the HS severity questionnaire validation study.

		Hurley Stage			
		I (Mild) (N = 39)	II (Moderate) (N = 43)	III (Severe) (N = 35)	All (N = 117)
Age at enrollment (Years)	Mean (SD)	36.7 (13.5)	39.6 (13.7)	42.3 (14.1)	39.4 (13.8)
	Median	32.4	35.4	44.0	36.1
	Min, Max	19.3, 68.7	19.6, 71.8	18.1, 70.5	18.1, 71.8
Gender	Male	12 (30.8%)	14 (32.6%)	13 (37.1%)	39 (33.3%)
	Female	27 (69.2%)	29 (67.4%)	22 (62.9%)	78 (66.7%)
Duration of HS Symptoms	Less than 12 months	3 (7.7%)	2 (4.7%)	0 (0.0%)	5 (4.3%)
	12 months to 5 years	15 (38.5%)	6 (14.0%)	4 (11.4%)	25 (21.4%)
	5 to 10 years	9 (23.1%)	10 (23.3%)	5 (14.3%)	24 (20.5%)
	10 years or longer	12 (30.8%)	25 (58.1%)	26 (74.3%)	63 (53.8%)
Length of HS Time since HS diagnosis	Less than 12 months	14 (35.9%)	3 (7.0%)	4 (11.4%)	21 (17.9%)
	12 months to 5 years	10 (25.6%)	19 (44.2%)	6 (17.1%)	35 (29.9%)
	5 to 10 years	11 (28.2%)	6 (14.0%)	7 (20.0%)	24 (20.5%)
	10 years or longer	4 (10.3%)	15 (34.9%)	18 (51.4%)	37 (31.6%)
Total DLQI scores	Mean (SD)	10.3 (7.7)	10.5 (7.9)	15.0 (8.2)	11.8 (8.1)
	Median	9.0	8.0	12.0	10.0
	Min, Max	0.0, 28.0	0.0, 28.0	2.0, 30.0	0.0, 30.0

The mean age of the study population was 39±14 years and two-thirds of patients (66.7%) were female (Table 2).

Approximately half of the patients (53.8%) had experienced HS symptoms for 10 or more years, however only 31.6% of patients had been diagnosed with HS for 10 or more years and patients with more severe disease appeared to have had HS symptoms for a longer period of time (Table 2). The mean DLQI score 11.8±8.1 (i.e. very large effect on quality of life) in the overall population, mean scores by Hurley Stage are presented in Table 2.

Table 3 reports HS treatments received by the study patients in the past or at the time of the study visit.

**Table 3:** Combined *past and current* treatments for HS by Hurley Stage in patients enrolled in the HS severity questionnaire validation study.

	Hurley Stage			
	I (Mild) (N = 39)	II (Moderate) (N = 43)	III (Severe) (N = 35)	All (N = 117)
Oral antibiotics	36 (92.3%)	42 (97.7%)	34 (97.1%)	112 (95.7%)
Topical antibiotics	20 (51.3%)	30 (69.8%)	26 (74.3%)	76 (65.0%)
Isotretinoin (oral medication)	10 (25.6%)	20 (46.5%)	23 (65.7%)	53 (45.3%)
Surgery	10 (25.6%)	17 (39.5%)	19 (54.3%)	46 (39.3%)
Oral contraceptive pill	11 (28.2%)	13 (30.2%)	12 (34.3%)	36 (30.8%)
Other oral medication or injection	10 (25.6%)	9 (20.9%)	11 (31.4%)	30 (25.6%)
Adalimumab	2 (5.1%)	11 (25.6%)	10 (28.6%)	23 (19.7%)
Clinical trial of a biologic	1 (2.6%)	10 (23.3%)	4 (11.4%)	15 (12.8%)
Acitretin (oral medication)	2 (5.1%)	2 (4.7%)	6 (17.1%)	10 (8.5%)
Infliximab	0 (0.0%)	3 (7.0%)	4 (11.4%)	7 (6.0%)
Etanercept	0 (0.0%)	1 (2.3%)	0 (0.0%)	1 (0.9%)
Ustekinumab	0 (0.0%)	0 (0.0%)	0 (0.0%)	0 (0.0%)

Almost all patients had been treated with oral antibiotics at some stage and independently of the severity of their disease, and more than half of them had used topical treatments. Biologics, isotretinoin, and surgery treatments had mostly been used in Hurley Stage II and III patients. Specifically 39.5% of Hurley stage II and 54.3% of Hurley stage III patients had received surgery. Acitretin use was relatively low (8.5% overall) and had more commonly been used to treat Stage III patients (17.1%) compared to Stage I (5.1%) or Stage II (4.7%) patients.

The most common past treatments for HS were oral antibiotics (48.7%), topical antibiotics (41.9%), and surgery (35.9%) (S3 Table, Supporting Information). The most common current treatments for HS were oral antibiotics (47%), topical antibiotics (23.1%) and oral contraceptive pills (16.2%) (S3 Table, Supporting Information).

#### Impact of the disease on HS patients

Almost all enrolled patients (113/117) answered every question of the HS severity questionnaire. Results are shown in the supporting information (S4 Table, Supporting Information). The majority of patients (59.8%) had experienced at least 4 sore or painful boils in the last 6 months (S4 Table, Question 1), and many (87.2%) reported boils or lumps leaving scars harder than the skin around the scars, at some stage in their lives (S4 Table, Question 3). Boils or scars in the last 6 months had often restricted the patients’ movements (82.9%, S4 Table, Question 4a), interfered with their work/school activities (65.0%, S4 Table, Question 4b), caused embarrassment or shame (79.5%, S4 Table, Question 4c), and impacted on personal or physical relationships (65.8%, S4 Table, Question 4d).

Approximately half of study patients (52.1%) rated their maximum pain as 8 out of 10 or higher in the last 6 months. The mean pain score reported by the overall study population was 6.6±2.8 (S4 Table, Question 2).

The overall impact of HS on the study patients’ quality of life is confirmed by the DLQI scores: for about half of patients (49.6%), HS had a very large or extremely large effect on their quality of life (Table 4).

**Table 4:** Impact of HS on quality of life in patients enrolled in the HS severity questionnaire validation study.

DLQI scores and associated meaning	All enrolled population (N = 117)
No effect at all on patient’s life (0–1)	7 (6.0%)
Small effect on patient’s life (2–5)	25 (21.4%)
Moderate effect on patient’s life (6–10)	27 (23.1%)
Very large effect on patient’s life (11–20)	33 (28.2%)
Extremely large effect on patient’s life (21–30)	25 (21.4%)

#### Ability of the HS severity questionnaire to predict Hurley Stage

The experimental HS severity questionnaire included eight questions encompassing aspects such as the presence and number of painful boils in the last 6 months, presence and size of scarring and impact of boils and/or scars on pain, movements, daily activities, relationships, and embarrassment. The full questionnaire is reported in Table 1.

Results from the multinomial logistic regression model showed that in the univariate analyses, only two questions in the HS severity questionnaire had a statistically significant association with Hurley Stage (p<0.05) (Table 5). Patients who answered “Yes” to “In the last 6 months, have the boils or scars interrupted your work/school activities?”, had a 3.5 times greater odds of being Hurley Stage III than stage I (OR Stage III vs Stage I = 3.5, 95% CI 1.2–10.3, p = 0.0247) (Question 4b, Table 5). In addition, patients with an area of “ugly/distressing” scarred skin of greater than 2 hands had a 2.7 times greater odds of being Hurley Stage III than Stage I (OR Stage III vs Stage I = 2.7, 95% CI 1.0–6.8, p = 0.0418) (Question 7, Table 5).

**Table 5:** Association between individual questions from the HS severity questionnaire and Hurley Stage of moderate versus mild, and severe versus mild.

Questions from the HS severity questionnaire	Effect	Hurley Stage	Univariate ^a^			Multivariate ^b^		
			Odd Ratio	95% CI	p-value	Odd Ratio	95% CI	p-value
1: In the last 6 months, how many sore or painful boils/lumps at least 1 cm (or half an inch) in diameter have you had? ^c^	1 unit increase	Moderate vs Mild	1.03	(0.88, 1.21)	0.7108	1.03	(0.80, 1.32)	0.8464
		Severe vs Mild	1.05	(0.89, 1.25)	0.5379	0.87	(0.66, 1.14)	0.3072
2: In the last 6 months, what is the most pain you have experienced from your boils or lumps (0 is no pain and 10 is unbearable pain)?	1 unit increase	Moderate vs Mild	0.89	(0.76, 1.05)	0.1618	0.84	(0.65, 1.08)	0.1680
		Severe vs Mild	0.98	(0.82, 1.17)	0.8132	0.86	(0.65, 1.13)	0.2714
3: Have you ever had boils or lumps heal leaving scars that feel harder than the skin around the scars? (at any stage in your life, not just the last 6 months)	Yes vs No	Moderate vs Mild	1.66	(0.48, 5.75)	0.4218	1.10	(0.25, 4.84)	0.9035
		Severe vs Mild	2.33	(0.55, 9.83)	0.2483	2.87	(0.50, 16.54)	0.2371
4a: In the last 6 months, have the boils or scars restricted your movements?	Yes vs No	Moderate vs Mild	0.60	(0.20, 1.84)	0.3719	0.46	(0.09, 2.38)	0.3557
		Severe vs Mild	1.88	(0.43, 8.17)	0.4005	1.17	(0.16, 8.38)	0.8722
4b: In the last 6 months, have the boils or scars interfered with your work/school activities?	Yes vs No	Moderate vs Mild	1.30	(0.54, 3.16)	0.5566	2.44	(0.70, 8.48)	0.1602
		Severe vs Mild	3.48	(1.17, 10.32)	0.0247	5.76	(1.31, 25.32)	0.0205
4c: In the last 6 months, have the boils or scars caused you embarrassment or shame?	Yes vs No	Moderate vs Mild	1.77	(0.60, 5.24)	0.2996	3.42	(0.81, 14.45)	0.0941
		Severe vs Mild	1.61	(0.52, 5.02)	0.4124	0.95	(0.19, 4.78)	0.9533
4d: In the last 6 months, have the boils or scars impacted on your personal or physical relationships?	Yes vs No	Moderate vs Mild	0.56	(0.23, 1.39)	0.2130	0.32	(0.09, 1.18)	0.0868
		Severe vs Mild	1.44	(0.51, 4.10)	0.4899	0.84	(0.18, 4.00)	0.8264
5: Does the skin of the area affected ever heal completely (possibly leaving scars)?	Yes vs No	Moderate vs Mild	0.81	(0.34, 1.93)	0.6343	0.83	(0.31, 2.26)	0.7159
		Severe vs Mild	0.42	(0.16, 1.09)	0.0735	0.42	(0.14, 1.26)	0.1212
6: Does the area affected by the scarring ever feel “lumpy” or the contour of “bubble wrap”?	Yes vs No	Moderate vs Mild	2.13	(0.57, 7.94)	0.2590	3.04	(0.64, 14.44)	0.1629
		Severe vs Mild	1.31	(0.38, 4.58)	0.6707	1.32	(0.28, 6.21)	0.7235
7: How big is the area currently affected by scarring that you would regard as ugly/distressing?	> 2 vs ≤ 2	Moderate vs Mild	1.58	(0.65, 3.88)	0.3155	1.71	(0.53, 5.51)	0.3694
		Severe vs Mild	2.67	(1.04, 6.85)	0.0418	2.35	(0.66, 8.37)	0.1856
8: Does the area currently affected always contain some painful pus-filled boils, lumps and scars?	Yes vs No	Moderate vs Mild	1.30	(0.47, 3.64)	0.6141	1.46	(0.42, 5.09)	0.5534
		Severe vs Mild	3.68	(0.92, 14.69)	0.0652	2.76	(0.53, 14.28)	0.2252

a Univariate results are from a multinomial logistic regression model with Hurley Stage as the outcome, and individual questions as the predictor variable.

b Multivariate results are from a multinomial logistic regression model with Hurley Stage as the outcome, and questions 1 to 8 as the predictor variables

c Due to small frequencies in some categories, question 1 was modelled as a continuous variable by mapping response 0 to 0, 1 to 1, 2–3 to 2.5, 4–6 to 5, and >6 to 8.

However, after adjusting for all the other questions in a multivariate analysis, only the association between Question 4b and Hurley Stage was still apparent (p = 0.0205). Patients who answered “Yes” to “In the last 6 months, have the boils or scars interrupted your work/school activities?” had a 5.8 times greater odds of being Hurley Stage III than Stage I (OR Stage III vs Stage I = 5.8, 95% CI 1.3 to 25.3, p = 0.0205) (Table 5).

The results were similar using logistic regression to model “mild” versus “moderate” or “severe”. In the univariate analyses, there was no statistically significant association between any question and Hurley Stage and in the multivariate analysis there was no statistically significant association between any question and Hurley Stage except for Question 4b, where patients who answered “Yes” to Question 4b were 3.18 times more likely to be Hurley Stage severe/moderate compared to mild (OR severe vs mild = 3.18, 95% CI 1.04 to 9.71, p-value = 0.0427).

Similarly, using logistic regression to model “severe” versus “mild” or “moderate”, there was no statistically significant association between any question and Hurley Stage, except for Question 4b, where patients who answered “Yes” were 3.03 times more likely to be Hurley Stage “severe” compared to “mild” or “moderate” (OR severe vs mild/moderate = 3.03, 95% CI 1.13 to 8.14, p-value = 0.0279). In the multivariate analysis the association between Question 4b and Hurley Stage was close to statistically significant (p-value = 0.0557), where patients who answered “Yes” to Question 4b were 3.66 times more likely to be Hurley Stage “severe” compared to “mild” or “moderate” (OR severe vs mild/moderate = 3.66, 95% CI 0.97 to 13.84).

For the HS severity questionnaire, the agreement between the true Hurley stage and the prediction from the models was only fair (Kappa ranged from 0.31 to 0.35). The prediction of moderate/severe patients versus mild had reasonable SE (80% to 91%) and PPV (73% to 79%), but poor SP (32% to 57%) and NPP (58% to 63%). The prediction of severe patients versus mild/moderate had poor SE (27% to 55%) and PPV (45% to 64%) but reasonable SP (73% to 94%) and NPP (76% to 79%).

When analysing the ability of the HS severity questionnaire to predict Hurley Stage, results from the three statistical models were consistent (Table 6). The ability of overall DLQI score to predict Hurley Stage as also consistent with the questionnaire.

**Table 6:** Predictive ability, sensitivity and specificity of the experimental self-administered HS severity questionnaire and DLQI against the Hurley Stage.

Hurley Categories ^a^	Model	Agreement (κ)	SE ^b^	SP ^b^	PPV ^b^	NPV ^b^
Mild, Moderate, Severe	Multinomial Logistic	0.35	n/a	n/a	n/a	n/a
	Canonical Discriminant Analysis	0.31	n/a	n/a	n/a	n/a
	DLQI Scores ^c^	0.17	n/a	n/a	n/a	n/a
Severe/Moderate versus Mild	Multinomial Logistic	n/a	0.80	0.57	0.79	0.58
	Logistic Regression	n/a	0.91	0.32	0.73	0.63
	Canonical Discriminant Analysis	n/a	0.82	0.54	0.78	0.59
	DLQI Scores ^c^	n/a	0.78	0.38	0.72	0.47
Severe versus Mild/Moderate	Multinomial Logistic	n/a	0.48	0.80	0.50	0.79
	Logistic Regression	n/a	0.27	0.94	0.64	0.76
	Canonical Discriminant Analysis	n/a	0.55	0.73	0.45	0.79
	DLQI Scores ^c^	n/a	0.66	0.57	0.40	0.80

a Hurley Stage I = Mild, Hurley Stage II = Moderate, Hurley Stage III = Severe

b Sensitivity (SE), Specificity (SP), Positive Predictive Value (PPV) and Negative Predictive Value (NPV)

c The overall DLQI score was used to predict Hurley Stage, where patients with a total DLQI score of 0–5 were predicted to be Hurley Stage I, 6–10 were predicted to be Stage II, and 11–30 were predicted to be stage III.

The overall DLQI scores showed only slight agreement with the Hurley Stage (Kappa = 0.17) (Table 6). Using DLQI overall scores for the prediction of moderate/severe patients versus mild had reasonable SE (78%) and PPV (72%) but poor SP (38%) and NPV (47%). The prediction of severe patients versus mild/moderate had low SE (66%), PPV (40%), and SP (57%) but a reasonable NPV (80%).

### II. HS Epidemiology in Australia

A population-based cross-sectional study was performed to assess HS prevalence and diagnosis rate in the Australian adult population as well as demographics and management pathways of individuals living with HS.

#### Study population

Out of the 17,050 Australian residents aged ≥18 years old interviewed by Roy Morgan Research, 11,433 agreed to answer the HS questionnaire. Gender distribution of the non-respondent population was similar to that of the respondent population (p = 0.29). Differences in terms of age and household distribution were statistically significant (p = 0.0061 and p<0.001 respectively) but not large (Table 7).

**Table 7:** Profile^a^ of Non-participants^a^ versus participants.

		Non-participants (N = 5,617)^a^	Participants (N = 11,433)^a^	p-value
Gender	Male	2,808 (50.0%)	5,616 (49.1%)	0.29
	Female	2,809 (50.0%)	5,817 (50.9%)	
Age category	18–24 years	497 (8.8%)	1,042 (9.1%)	0.0061
	25–34 years	880 (15.7%)	1,636 (14.3%)	
	35–49 years	1,233 (22.0%)	2,577 (22.5%)	
	50–64 years	1,343 (23.9%)	2,961 (25.9%)	
	65 years & over	1,664 (29.6%)	3,217 (28.1%)	
Household location ^b^	VIC	1,401 (24.9%)	2,758 (24.1%)	< .001
	NSW/ACT	1,814 (32.3%)	3,894 (34.1%)	
	QLD	1,166 (20.8%)	2,230 (19.5%)	
	SA	503 (9.0%)	926 (8.1%)	
	WA	508 (9.0%)	1,262 (11.0%)	
	NT/TAS	225 (4.0%)	363 (3.2%)	

^a^ PARTICIPANTS: Approached Australian residents aged ≥18yo who agreed to answer the HS questionnaire. NON-PARTICIPANTS: Individuals interviewed by Roy Morgan Research who refused to answer the HS questionnaire.

^b^ VIC = Victoria, NSW = New South Wales, ACT = Australian Capital Territory, QLD = Queensland, SA = South Australia, WA = Western Australia, NT = Northern Territory and TAS = Tasmania.

The characteristics of the respondent population (N = 11,433, Table 7) appeared generally consistent with the Australian data reported by the Australian Bureau of Statistics [22], a standard reference source for Australian population demographics. A sensitivity analysis performed after post-stratifying our sample to reflect the exact age and sex distribution of the Australian population gave similar results. These results do not indicate a reason to suspect that our respondent population is not representative as the Australian population.

#### HS prevalence in the Australian adult population

Based on the results from the HS screening questionnaire, 88 out of the 11,433 respondents were identified as potentially having HS (0.77%; 95% CI 0.62 to 0.95) (Table 8). Data by State are reported in Table 8 and a sensitivity analysis adjusted for stratification by state yielded the same point estimate and confidence intervals.

**Table 8:** Suspected HS cases by state and national total.

State ^a^	Total number of people interviewed ^b^	Individuals answering HS questions	Suspected HS cases ^c^	
			n	% (95% CI)
**VIC**	4159	2758	26	0.94% (0.62%; 1.38%)
**NSW / ACT**	5708	3894	24	0.62% (0.40%; 0.92%)
**QLD**	3396	2230	14	0.63% (0.34%; 1.05%)
**SA**	1429	926	11	1.19% (0.59%; 2.12%)
**WA**	1770	1262	9	0.71% (0.33%; 1.35%)
**NT**	107	51	2	3.92% (0.48%; 13.46%)
**TAS**	481	312	2	0.64% (0.08%; 2.30%)
**TOTAL**	**17050**	**11433**	**88**	**0.77% (0.62%; 0.95%)**

^a^ VIC = Victoria, NSW = New South Wales, ACT = Australian Capital Territory, QLD = Queensland, SA = South Australia, WA = Western Australia, NT = Northern Territory and TAS = Tasmania.

^b^ Australian residents participating in the Single Source establishment survey run by Roy Morgan Research.

^c^ Based on the HS screening questionnaire (S1 Table).

Considering the previously reported sensitivity and positive predictive value of the screening questionnaire [13], the prevalence of HS was estimated to be 0.67% (95% CI 0.53% to 0.84%) (Table 9). HS appeared to be twice as prevalent in females compared to males (estimated HS prevalence after adjusting for SE and PPV of the screening question: 0.88% [95% CI 0.67–1.15] in females vs 0.46% [95% CI 0.32–0.68] in males, p = 0.0068) (Table 9).

**Table 9:** Estimated prevalence of HS in Australia overall and by gender.

		Overall (N = 11433)	Females (N = 5817)	Males (N = 5616)	Chi-square p-value ^c^
Suspected HS cases ^a^	n/N (%) [95% Cl]	88/11433 (0.77%) [0.62%; 0.95%]	58/5817 (1.0%) [0.77;1.29]	30/5616 (0.53%) [0.36;0.76]	0.0046
Estimated HS Prevalence ^b^	n/N (%) [95% Cl]	77/11433 (0.67%) [0.53; 0.84]	51/5817 (0.88%) [0.67; 1.15]	26/5616 (0.46%) [0.32; 0.68]	0.0068

^a^ Based on the HS screening questionnaire. A suspected HS subject is a subject with a presence of outbreaks AND at least one boil location other than «Other Locations».

^b^ Based on Sensitivity and Positive Predictive Value of the HS screening questionnaire (Sensitivity 0.97, Positive Predictive Value 0.85) [13]

^c^ Chi-square test to compare results in Females versus Males

In women, boils were most commonly reported in the groin area (56.9%) and armpits (44.8%), followed by under the breasts (25.9%), sexual organs (20.7%), anal region (19%), and folds on the stomach/around the navel (17.2%) (Table 10). Men most commonly reported boils in the groin area (43.3%) and anal region (36.7%), followed by armpits (30%), folds on the stomach/around the navel (26.7%), sexual organs and under the breasts (both 6.7%) (Table 10).

**Table 10:** Location of boils in suspected HS individuals, total and by gender.

Location of boils ^a^	Total (N = 88)	Females (N = 58)	Males (N = 30)	Chi-square p-value ^b^
Groin	46/88 (52.3%)	33/58 (56.9%)	13/30 (43.3%)	0.2273
Armpits	35/88 (39.8%)	26/58 (44.8%)	9/30 (30.0%)	0.1779
Sexual organs	14/88 (15.9%)	12/58 (20.7%)	2/30 (6.7%)	0.0882
Anal region	22/88 (25.0%)	11/58 (19.0%)	11/30 (36.7%)	0.0691
Under the breasts	17/88 (19.3%)	15/58 (25.9%)	2/30 (6.7%)	0.0306
Folds on the stomach / around the navel	18/88 (20.5%)	10/58 (17.2%)	8/30 (26.7%)	0.2988

^a^ A subject can have reported more than one boil location.

^b^ Chi-square test to compare results in Females versus Males

People living with HS in Australia: characteristics, diagnosis rate and management pathways.

The diagnosis rate among the individuals who screened positive for HS was low, with only 6 out of the 88 individuals identified through the HS screening questionnaire having reported a previous diagnosis of HS or *acne inversa* (6.8%; 95% CI 3.2% to 14.1%).

Among the individuals with a previous diagnosis of HS (N = 6), half (3/6) had been diagnosed by a dermatologist, 2/6 by a GP, 1/6 by an infectious disease specialist. A third (2/6) had seen 5 or more clinicians regarding their condition before receiving a diagnosis. Among the undiagnosed individuals suspected of having HS (N = 82), a quarter (21/82, 25.6%) had not seen any clinicians regarding their boils, and the remainder had consulted General Practitioners (59/61, 96.7%) and specialists, including dermatologists (12/61, 19.7%), surgeons (7/61, 11.5%) and infectious disease specialists (5/61, 8.2%) as shown in Supporting information S5 Table.

A post-hoc analysis of demographic characteristics in suspected HS individuals (N = 88) compared with non-HS individuals (N = 11,345) (Table 11) demonstrated a statistically significant association between HS status and gender (p = 0.0046), age (p<0.0001), BMI (p = 0.0307), smoking status (p<0.0001), employment status (p<0.0001) and income (p = 0.0321). Individuals suspected of having HS were more likely to be females, young, obese, smokers, unemployed or at home duties and having a lower annual personal income in comparison with non-HS individuals (Table 11).

**Table 11:** Characteristics of people living with HS in Australia compared to non-HS.

		Not-HS (N = 11345)	Suspected HS^a^ (N = 88)	Total (N = 11433)	p-value
Gender	Male	49.2%	34.1%	49.1%	0.0046
	Female	50.8%	65.9%	50.9%	
Age category	18–24 years	9.1%	6.8%	9.1%	< .0001
	25–34 years	14.3%	17.0%	14.3%	
	35–44 years	14.1%	33.0%	14.2%	
	45–54 years	16.6%	21.6%	16.7%	
	55–64 years	17.6%	14.8%	17.6%	
	65 years & over	28.3%	6.8%	28.1%	
Country of Birth	Australia	74.4%	86.4%	74.5%	0.0102
	Other	25.6%	13.6%	25.5%	

Household location	VIC	24.1%	29.5%	24.1%	0.3508
	NSW/ACT	34.1%	27.3%	34.1%	
	QLD	19.5%	15.9%	19.5%	
	SA	8.1%	12.5%	8.1%	
	WA	11.0%	10.2%	11.0%	
	NT/TAS	3.2%	4.5%	3.2%	
Smoker	No	83.9%	45.5%	83.6%	< .0001
	Yes	16.1%	54.5%	16.4%	
Highest education level	High-school	47.1%	48.9%	47.1%	0.7432
	University	52.9%	51.1%	52.9%	
Personal Annual Income	Less than 20K (AUD)	27.4%	22.7%	27.3%	0.0321
	20K-<40K (AUD)	27.4%	39.8%	27.5%	
	40K-<80K (AUD)	26.8%	27.3%	26.8%	
	80K(AUD) or more	18.5%	10.2%	18.4%	
Employment status	Employed	56.4%	45.5%	56.3%	< .0001
	Unemployed	8.2%	20.5%	8.3%	
	Retired	28.9%	17.0%	28.8%	
	Student	1.9%	2.3%	1.9%	
	Home duties	4.5%	14.8%	4.6%	
Occupation	Professional/Semi-pro/Sales	26.9%	27.5%	26.9%	0.2630
	Executive/White collar	37.2%	50.0%	37.3%	
	Skilled/Semi-skilled	28.2%	22.5%	28.2%	
	Unskilled/Farm owner/worker	7.0%	0	7.0%	
	Unclassified	0.6%	0	0.6%	
Marital status	Married/De Facto	57.7%	52.3%	57.6%	0.1526
	Single/Separated/Engaged/ Planning to marry	25.2%	34.1%	25.3%	
	Widowed/Divorced	17.1%	13.6%	17.1%	
BMI category	Underweight	1.4%	0	1.4%	0.0307
	Acceptable weight	29.9%	17.2%	29.8%	
	Overweight	34.1%	20.7%	34.0%	
	Obese	32.3%	55.2%	32.5%	
	Unclassified	2.3%	6.9%	2.4%	

^a^ Individuals suspected of having HS (N = 88) based on the results of the HS screening questionnaire (S1 Table).

#### Clinical assessment of suspected HS cases

Of the 88 Roy Morgan Single Source Survey participants identified as possibly having HS, 39 (44%) consented to share their contact details to schedule a clinical assessment at the closest dermatology clinic, but only 12 of them actually attended the visit at the dermatologist (Patients’ disposition is reported in the Supporting information, S6 Table).

The demographic characteristics of the attendees (N = 12) and non-attendees (N = 76) are shown in S7 Table. Some differences between these two groups could be observed in terms of age, income, gender distribution, BMI and pain. Among those who attended the clinic, 50% were men and 50% women (versus 32% men and 68% women in the non-attending group), 33% were 55 years old and over, and none of them were aged between 18-24yo (versus 20% 55 years old and 8% aged 18–24 years old in the non-attending group), 58% earned 40,000 AUD or more (versus 33% in the non-attending group), 42% earned less than 40,000 AUD (versus 67% in the non-attending group), 29% were obese (versus 61% in the non-attending group), 17% had reported a pain of 8 or more out of 10 (versus 39.5% in the non-attending group), and none of them reported a maximum pain of 10/10 (versus 16% in the non-attending group)

Following the clinical assessment, 7 out of 12 individuals had a confirmed diagnosis of HS (Table 12): 5 of them were Hurley Stage I and 2 Hurley Stage II. No Hurley Stage III cases were identified at the dermatology clinic. Only one of these cases had been previously diagnosed with HS, the remaining 6 cases were diagnosed for the first time during the clinical assessment, despite 4 of them having previously consulted at least one health care professional regarding their boils.

**Table 12:** Characteristics of the 7 confirmed cases of HS through physical examination among the 12 who attended the visit at the dermatologist.

		Hurley Stage			All (N = 7)
		I (N = 5)	II (N = 2)	III (N = 0)	
Clinical diagnosis for HS		5/7 (71%)	2/7 (29%)	0	7/7 (100%)
Gender	Male	1/5 (20%)	2/2 (100%)	0	3/7 (43%)
	Female	4/5 (80%)	0	0	4/7 (57%)
HS/Acne Inversa previously diagnosed		1/5 (20%)	0	0	1/7 (14%)
Duration of HS symptoms	Less than 12 months	0	0	0	0
	12 months up to 5 years ago	0	1/2 (50%)	0	1/7 (14%)
	5 years up to 10 years ago	2/5 (40%)	0	0	2/7 (29%)
	10 years or longer	3/5 (60%)	1/2 (50%)	0	4/7 (57%)
Health Problems in addition to HS		3/5 (60.0%)	2/2 (100.0%)	0	5/7 (71.4%)
List of Health Problems in addition to HS ^a^	Obesity	2/5 (40.0%)	0	0	2/7 (28.6%)
	Depression	2/5 (40.0%)	0	0	2/7 (28.6%)
	Crohn's disease	0	0	0	0
	Ulcerative colitis	0	0	0	0
	Spondyloarthropathy	0	0	0	0
	Pyoderma Gangrenosum	0	0	0	0
	Other	3/5 (60.0%)	2/2 (100.0%)	0	5/7 (71.4%)

a A subject can have answered more than one health problem in addition to HS. The denominator corresponds to the number of subjects who answered having a health problem in addition to HS

Most of the individuals with a confirmed HS diagnosis had experienced symptoms of HS for more than 5 years (6/7) and had concomitant health issues in addition to HS (5/7), including obesity (2/5) and depression (2/5) (Table 12).

The 5/12 individuals who did not receive a confirmed diagnosis of HS at the clinic visit, were diagnosed with: chronic folliculitis, epidermoid cyst affecting the back, psoriasis, recurrent furunculosis, ruptured acute folliculitis.

Of the 12 who attended the clinic visit, 10 had clinical photographs of their lesions taken. The two missing sets of photographs belonged to two non-confirmed cases of HS. All the lesions’ photographs were reviewed by an external reviewer with longstanding expertise in HS, who was blinded to the results of the physical assessment performed by the clinicians. The level of agreement was good (80%) (Table 13). The clinicians and expert reviewer agreed on 2 cases not corresponding to HS disease as well as on the 7 confirmed cases of HS (Table 13). However, the external reviewer identified an additional case not diagnosed as HS in the clinic as Hurley Stage I HS, bringing the number of confirmed HS cases to a total of 8 (Table 13). The clinician and the expert reviewer agreed on the severity assessment of 6 out of 7 cases (Table 13).

**Table 13:** Agreement between clinical and expert assessments in suspected HS individuals attending the dermatology visit (N = 12).

		Expert Severity Diagnosis			
	Missing	Diagnosed as not HS	Stage I	Stage II	Stage III
Clinical Severity Diagnosis					
Missing Photographs	2	0	0	0	0
Diagnosed as not HS	0	2	1	0	0
Stage I	0	0	5	0	0
Stage II	0	0	1	1	0
Stage III	0	0	0	0	0

## Discussion

### I. Validation of an experimental self-administered questionnaire to predict the severity of HS

A self-administered HS severity questionnaire (Table 1) was developed with the aim of providing a practical means of gauging HS severity distribution in a population-based epidemiology study.

Overall, the experimental HS severity questionnaire and DLQI were found to be better than chance at predicting Hurley Stage in HS patients, but not accurate enough to be an adequate prediction tool. These results could at least partially be explained by the limitations of the Hurley Staging system. Although considered to be the gold standard to assess grades of severity for each area of the body affected by HS and being routinely used in clinical practice to define appropriate treatment options, Hurley staging system cannot provide a global scoring of severity, doesn’t incorporate inflammatory and Quality of Life features and is based on static disease characteristics which can be irreversible, such as scarring [5, 15]. Of note, many study patients had received or were currently receiving treatment for HS, but improvement of their disease might have not been reflected in their current Hurley stage because of the static nature of this severity measure. Acknowledging the limitations of the Hurley Staging, efforts from groups of HS experts are underway to define a more detailed and suitable subclassification of the disease [23, 24]. Future studies could address the ability of the HS severity questionnaire, or part of it, to predict severity as defined by new severity classification methods.

DLQI is a 10-item survey evaluating the quality of life in patients with a skin disease, covering six domains: symptoms and feelings, daily activities, leisure, work and school, personal relationships and treatment [25]. While reduction in severity scores in dermatologic conditions, such as psoriasis, can correlate well with DLQI [26], it can be difficult to correlate absolute values of disease severity and DLQI given the subjectivity of the latter. In addition to the limitations of the Hurley staging discussed above, this could have also contributed to the observed lack of ability of DLQI to predict Hurley Stage in our cross-sectional study.

Among the 8 questions included in the experimental HS severity questionnaire (Table 1), interference of the boils or scars with work/school activities and the area of ugly/distressing scarred skin in HS patients were associated with Hurley stage in the univariate analyses. However, the association with the area of scarred skin was not statistically significant in the multivariate analyses. These data could be leveraged for the future development of self-administered tools to assess HS severity.

Despite the HS severity questionnaire and DLQI lacking the ability to accurately predict Hurley Stage in HS patients, our data provided insights into the burden of the disease. The overall DLQI scores reported in our study demonstrated that HS had a large or extremely large effect on quality of life for nearly 50% of the study patients with a mean DLQI score of 11.8±8.1 in the overall study population. Study patients were also suffering from considerable pain, with approximately half of them rating their maximum pain as 8 out of 10 or higher in the last 6 months. These data are not surprising given the nature, localization and chronicity of HS lesions and are in agreement with the body of literature reporting that the impact of HS on quality of life is higher than in many other debilitating diseases (with a DLQI ranging from a mean of 8.4 to 20) [27, 28] and correlates with pain [29].

Interestingly, patients with more severe disease appeared to have had HS symptoms for a longer period of time. This is consistent with a progressive nature of the disease as suggested by previous reports showing that over time, the inflammatory lesions affect more areas of the body and grow in both size and number [17, 30, 31].

### II. HS Epidemiology in Australia

Based on face-to-face household interviews of a large (N = 11,433) representative sample of the adult Australian population using a previously validated HS screening questionnaire (S1 Table) [13], the prevalence of HS in Australia was estimated to be 0.67% (95% CI 0.53%-0.84%) after adjusting for the previously reported sensitivity and positive predictive value of the screening questionnaire [13].

Several studies have previously attempted to determine HS prevalence in different settings and using different methods. The result is a set of highly variable estimations of HS prevalence, ranging between 0.03% and 4% of the population, with the majority of the studies being small and/or from selected populations [6]. However, prevalence estimates coming from cross-sectional, population-based studies using screening questions and/or clinical assessment, like our study, are usually considered to be the most reliable. To our knowledge, only 3 studies have so far investigated HS prevalence in a population-based sample [32–34], and only 1 of them used a validated screening questionnaire [32], while the others used a questionnaire that had not been validated [33, 34]. With the former study, Vinding and colleagues [32] found a prevalence of 2.1% (95% CI 1.88–2.32) in the Danish population aged 30 years old and over. Data were obtained from the Danish General Suburban Population Study (GESUS), a general cross-sectional population study of the health status of the population in Naestved Municipality. The following factors could at least partially explain the different prevalence rates observed in this study and the Danish one: (I) it is unknown how much Naestved Municipality is representative of the entire Danish population, while the sample used in our study was representative of the Australian population including rural and urban areas; (II) participants were invited to participate to the GESUS health study by mail, the participation rate was therefore relatively low (49%) and a large number of participants (769 out of a total of 17,454) were excluded due to missing data. These factors may have introduced a bias to the results. The response rate in our study was 67% and the demographic characteristics of the respondent population were similar to that of non-respondents and appeared generally consistent with the Australian general population data; (III) No data for adults aged less than 30 years old were included in the Danish study; (IV) As opposed to the approach described herein, the prevalence rate reported in the Danish study was not adjusted based on the SE and PPV of the HS screening questionnaire used; (V) prevalence estimates could fluctuate based on geography and ethnicity [35, 36]. In line with our findings, a recent study using a very large number of UK residents with research-standard medical records from a large database found a 0.77% HS prevalence[11].

HS in Australia appeared to be twice as prevalent in females compared to males, in agreement with previous studies reporting female:male ratios ranging from 2:1 to 5:1 [6, 35, 37]. Overall, boils were mainly reported in the groin and armpits, in agreement with the data reported from a recent Italian study [38], and there appeared to be a trend towards slightly different patterns in the location of boils between women and men, as previously suggested by other research groups [15, 18, 39, 40]. In our study, males reported the anal region to be the second most common location of boils, while in females the second most common location of boils was reported to be armpits. Not unexpectedly, boils under the breasts were significantly more prevalent in women compared to males.

Interestingly, the diagnosis rate amongst the suspected HS cases was low, with only 6.8% (95% CI 3.2% -14.1%) having reported previously receiving a diagnosis of HS/*acne inversa*. An average of 7.2 years between symptom onset and HS diagnosis and high frequency of undiagnosed and misdiagnosed cases have previously been reported [7, 35]. Out of the 7 HS cases confirmed at the dermatology clinic, 6 had had HS symptoms for more than 5 years. Delayed diagnosis is a significant problem in the management of HS, as it is likely to result in under-treatment and, as a consequence, disease progression and increased disability. In our study, most of the confirmed cases of HS (4/7) were not receiving any treatment for their condition. Given that a quarter of the undiagnosed individuals suspected of having HS had not seen any clinician regarding their boils and that many diagnosed and undiagnosed individuals had seen several clinicians/specialists regarding their condition, it can be speculated that the low diagnosis rate observed in the Australian general population may result from a combination of decentralization of care, lack of familiarity of some clinicians with the disease, and many patients not seeking medical help for single or infrequently recurring boils. This would indicate a need to increase awareness of the disease in Australia among both the broader medical community and the general population.

Here we showed that individuals living with HS identified through the screening questionnaire were more likely to be females, young, obese, smokers, unemployed or at home duties and having a lower annual personal income in comparison with non-HS individuals. Smoking and obesity are known risk factors for HS [28, 35, 41] and the mean age of HS onset is in the early 20s with a decline of prevalence after the age of 55 [35]. Although cross-sectional studies do not provide data to establish causality, it may be speculated that the patients’ disease-related disabilities may affect their ability to work and subsequently also have a socioeconomic impact.

Only 12 out of the 88 suspected HS individuals attended the visit at the dermatologist, with 10 of them having photographs of their lesions taken. 7/12 received a confirmed diagnosis of HS through physical assessment. An additional individual was diagnosed with HS through the review of photographs by a blinded independent HS expert, bringing the number of confirmed HS cases to 8/12 (67%). Most of the HS patients had Stage I disease, while none were identified with Stage III disease. Obesity and depression, which are known comorbid conditions in HS [28, 35], were noted even in this small number of cases.

The main limitation of this study was the small proportion of patients attending the clinical assessment, which can not be considered representative of the overall 88 individuals suspected of having HS. The challenges in travel time characteristic of Australia’s dispersed population may have contributed to the low attendance rate. Also, although the number of individuals attending the clinics was too small to draw any definitive conclusions, characteristics of the attendees compared to those who did not attend the clinic suggest that there might have been a self-selection bias enriching the attending population with false negatives, given that the characteristics of that group appeared to be dissimilar to the typical HS patient profile, in contrast to the characteristics observed in the group who did not attend the clinic. It could be speculated that individuals affected by HS could have had more difficulty traveling to the clinic. This speculation is supported by the fact no Hurley Stage III HS cases (who are more likely to be limited by the disease-related physical and psychological disabilities) were identified at the clinical assessment. The possible self-selection bias of people attending the clinical assessment further supports the benefit of using a validated screening questionnaire on a representative sample of the general population, as in our study, as opposed to using data from medical databases or patients under medical care.

The low and unrepresentative attendance to the clinical assessment does not affect the validity of the epidemiology data obtained through the population-based survey as the HS screening questionnaire had already been previously validated by Esmann and colleagues [13], however we were not able to provide a further confirmation of the sensitivity of the questionnaire.

Because the data obtained from the clinical examination can not be generalized and the experimental self-administered severity questionnaire did not predict Hurley Stage, it was not possible to report proportion of mild, moderate and severe disease in the Australian HS population.

To our knowledge, our study represents one of the most rigorous and largest general population-based HS epidemiological studies reported thus far, and for the first time sheds light on the prevalence and impact of HS in Australia as well as the demographic characteristics of the sufferers. It can be speculated that the diverse genetic background of the Australian population may support the generalizability of our findings to a broader global population.

## Supporting information

S1 Table HS screening questionnaire supported by a visual diagram (adapted from Esmann et al [13]). (PDF)

S2 Table Study questions included in the HS questionnaire following the HS screening questionnaire. (PDF)

S3 Table Past and current treatments for HS in patients enrolled in the HS severity questionnaire validation study. (PDF)

S4 Table Summary of HS severity questionnaire results. (PDF)

S5 Table Clinicians consulted by diagnosed and undiagnosed individuals suspected of having HS ^a^. (PDF)

S6 Table Patients’ disposition for Stage 2 (clinical assessment) of the HS Epidemiology study. (PDF)

S7 Table Demographic characteristics of suspected HS individuals ^a^: Attended vs not attended clinic. (PDF)
