# Supplementary material for: Hidradenitis Suppurativa (HS) prevalence, demographics and management pathways in Australia: A population-based cross-sectional study
Source: PLoS One. 2018 Jul 24;13(7):e0200683. doi: 10.1371/journal.pone.0200683 (PMC6057625; doi:10.1371/journal.pone.0200683)
Supplement: S1 Table — (PDF) [file pone.0200683.s001.pdf]

S1 Table. HS screening questionnaire supported by a visual diagram (adapted from Esmann et al (10)) [a,b,c].

Do you repeatedly have outbreaks of big sore or painful nodules or boils that heal with scars in any of these locations? (tick all that apply)
Groin
Armpits
Sexual organs
Anal Region
Under the breasts
Folds on the stomach/around the navel
Other Locations [c]

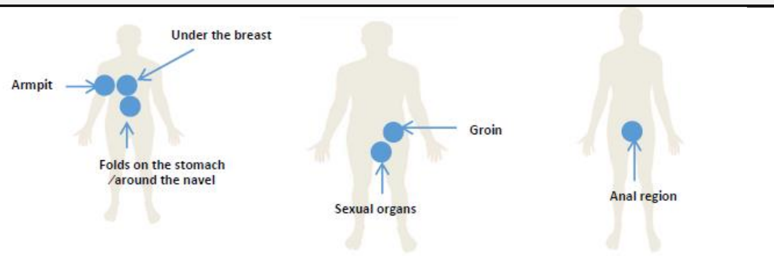

a Indirectly yes to boils: Sensitivity 0.97, Specificity 0.82, Positive Predictive Value 0.85 (10)
b In this study a diagram showing the locations listed in the table was also provided.
c The questionnaire used in this study also listed "Other Locations" but this was not used to identify possible HS patients (ie a suspected HS subject was an individual with presence of outbreaks AND at least one nodule/boil location other than "Other Locations")
