# Supplementary material for: Hidradenitis Suppurativa (HS) prevalence, demographics and management pathways in Australia: A population-based cross-sectional study
Source: PLoS One. 2018 Jul 24;13(7):e0200683. doi: 10.1371/journal.pone.0200683 (PMC6057625; doi:10.1371/journal.pone.0200683)
Supplement: S2 Table — (PDF) [file pone.0200683.s002.pdf]

S2 Table. Study questions included in the HS questionnaire following the HS screening questionnaire.

Have you been diagnosed with Hidradenitis Suppurativa or *Acne Inversa*?			
**YES**		**NO**	
From which clinician did you receive your diagnosis?	General practitioner	How many clinicians have you seen regarding your condition of boils?	1
	Dermatologist		2
	Surgeon		3
	Infectious disease specialist		4
	Gynaecologist		5
	Emergency specialist		More than 5
	Gastroenterologist		None
	Other		
How many clinicians did you see before you received your diagnosis?	1	Which clinician/s have you seen regarding your condition of boils (tick all that apply)?	General practitioner
	2		Dermatologist
	3		Surgeon
	4		Infectious disease specialist
	5		Gynaecologist
	More than 5		Emergency specialist
			Gastroenterologist
			Other
Which clinicians did you see prior to your diagnosis (tick all that apply)?	General practitioner	Which clinicians do you currently see regarding your condition of boils?	General practitioner
	Dermatologist		Dermatologist
	Surgeon		Surgeon
	Infectious disease specialist		Infectious disease specialist
	Gynaecologist		Gynaecologist
	Emergency specialist		Emergency specialist
	Gastroenterologist		Gastroenterologist
	Other		Other
			None
