# Supplementary material for: Hidradenitis Suppurativa (HS) prevalence, demographics and management pathways in Australia: A population-based cross-sectional study
Source: PLoS One. 2018 Jul 24;13(7):e0200683. doi: 10.1371/journal.pone.0200683 (PMC6057625; doi:10.1371/journal.pone.0200683)
Supplement: S3 Table — (PDF) [file pone.0200683.s003.pdf]

S3 Table. Past and Current Treatments for HS in patients enrolled in the HS severity questionnaire validation study.

Treatment	Past (N=117)	Current (N=117)
Oral antibiotics	57 (48.7%)	55 (47.0%)
Topical antibiotics	49 (41.9%)	27 (23.1%)
Isotretinoin (oral medication)	42 (35.9%)	19 (16.2%)
Surgery	39 (33.3%)	16 (13.7%)
Oral contraceptive pill	17 (14.5%)	16 (13.7%)
Other oral medication or injection	14 (12.0%)	14 (12.0%)
Adalimumab	8 (6.8%)	12 (10.3%)
Clinical trial of a biologic	7 (6.0%)	5 (4.3%)
Acitretin (oral medication)	3 (2.6%)	4 (3.4%)
Infliximab	2 (1.7%)	2 (1.7%)
Etanercept	0 (0.0%)	1 (0.8%)
Ustekinumab	0 (0.0%)	0 (0.0%)
