# Supplementary material for: Hidradenitis Suppurativa (HS) prevalence, demographics and management pathways in Australia: A population-based cross-sectional study
Source: PLoS One. 2018 Jul 24;13(7):e0200683. doi: 10.1371/journal.pone.0200683 (PMC6057625; doi:10.1371/journal.pone.0200683)
Supplement: S4 Table — (PDF) [file pone.0200683.s004.pdf]

S4 Table. Summary of HS Severity Questionnaire Results.

	All Enrolled Population (N=117)
Q1. In the last 6 months, how many sore or painful boils/lumps at least 1 cm (or half an inch) in diameter have you had?	
0	6 (5.13%)
1	8 (6.84%)
2-3	31 (26.50%)
4-6	27 (23.08%)
> 6	43 (36.75%)
Q2. In the last 6 months what is the most pain you have experienced from your boils or lumps (0 is no pain and 10 is unbearable pain)?	
n	116
Mean (SD)	6.60 (2.78)
SE	0.26
Median	8.00
Min, Max	0.0, 10.0
0	5 (4.27%)
1	4 (3.42%)
2	1 (0.85%)
3	9 (7.69%)
4	8 (6.84%)
5	9 (7.69%)
6	11 (9.40%)
7	8 (6.84%)
8	30 (25.64%)
9	16 (13.68%)
10	15 (12.82%)
Q3. Have you ever had boils or lumps heal leaving scars that feel harder than the skin around the scars? (at any stage in your life, not just the last 6 months)	
Yes	102 (87.18%)
No	15 (12.82%)
Q4. In the last 6 months, have the boils or scars:	
(a) Restricted your movements	
Yes	97 (82.91%)
No	19 (16.24%)
(b) Interfered with your work/school activities?	
Yes	76 (64.96%)
No	39 (33.33%)
(c) Caused you embarrassment or shame?	
Yes	93 (79.49%)
No	23 (19.66%)
(d) Impacted on personal or physical relationships?	
Yes	77 (65.81%)
No	39 (33.33%)

	All Enrolled Population (N=117)
Q5. Does the skin of the area affected ever heal completely (possibly leaving scars)?	
Yes	56 (47.86%)
No	60 (51.28%)
Q6. Does the area affected by scarring ever feel “lumpy” or the contour of “bubble wrap”?	
Yes	101 (86.32%)
No	16 (13.68%)
Q7. How big is the area currently affected by scarring that you would regard as ugly/distressing?	
Surface area of 3 hands or larger	39 (33.33%)
Surface area of less than 3 hands but more than 2 hands	13 (11.11%)
Surface area of 2 hands or less	65 (55.56%)
Q8. Does the area currently affected always contain some painful pus–filled boils, lumps and scars?	
Yes	95 (81.20%)
No	22 (18.80%)
