# Supplementary material for: Hidradenitis Suppurativa (HS) prevalence, demographics and management pathways in Australia: A population-based cross-sectional study
Source: PLoS One. 2018 Jul 24;13(7):e0200683. doi: 10.1371/journal.pone.0200683 (PMC6057625; doi:10.1371/journal.pone.0200683)
Supplement: S5 Table — (PDF) [file pone.0200683.s005.pdf]

S5 Table. Clinicians consulted by diagnosed and undiagnosed individuals suspected of having HS [a].

Have you been diagnosed with Hidradenitis Suppurativa or Acne Inversa?					
Yes<br>6/88 (6.8%)			No<br>82/88 (93.2%)		
From which clinician did you receive your diagnosis?	General practitioner	2/6 (33.3%)	How many clinicians have you seen regarding your condition of boils?	1	21/82 (25.6%)
	Dermatologist	3/6 (50.0%)		2	28/82 (34.1%)
	Surgeon	0		3	13/82 (15.9%)
	Infectious disease specialist	1/6 (16.7%)		4	4/82 (4.9%)
	Gynaecologist	0		5	3/82 (3.7%)
	Emergency specialist	0		More than 5	3/82 (3.7%)
	Gastroenterologist	0		None	10/82 (12.2%)
	Other	0			
How many clinicians did you see before you received your diagnosis?	1	0	Which clinician/s have you seen regarding your condition of boils (tick all that apply)? [b, c]	General practitioner	59/61 (96.7%)
	2	2/6 (33.3%)		Dermatologist	12/61 (19.7%)
	3	2/6 (33.3%)		Surgeon	7/61 (11.5%)
	4	0		Infectious disease specialist	5/61 (8.2%)
	5	0		Gynaecologist	2/61 (3.3%)
	More than 5	1/6 (16.7%)		Emergency specialist	3/61 (4.9%)
		1/6 (16.7%)		Gastroenterologist	1/61 (1.6%)
				Other	61/61 (100%)
Which clinicians did you see prior to your diagnosis (tick all that apply)? [c]	General practitioner	4/6 (66.7%)	Which clinicians do you currently see regarding your condition of boils? [b, c]	General practitioner	44/61 (72.1%)
	Dermatologist	1/6 (16.7%)		Dermatologist	0
	Surgeon	0		Surgeon	2/61 (3.3%)
	Infectious disease specialist	2/6 (33.3%)		Infectious disease specialist	2/61 (3.3%)
	Gynaecologist	0		Gynaecologist	1/61 (1.6%)
	Emergency specialist	1/6 (16.7%)		Emergency specialist	0
	Gastroenterologist	0		Gastroenterologist	0
	Other	6/6 (100%)		Other	2/61 (3.3%)
				None	15/61 (24.6%)

a Individuals suspected of having HS (N=88) based on the results of the HS screening questionnaire (Table S1)
b With at least one clinician seen regarding the condition of boils.
c A subject can have answered more than one clinician
