# Supplementary material for: Hidradenitis Suppurativa (HS) prevalence, demographics and management pathways in Australia: A population-based cross-sectional study
Source: PLoS One. 2018 Jul 24;13(7):e0200683. doi: 10.1371/journal.pone.0200683 (PMC6057625; doi:10.1371/journal.pone.0200683)
Supplement: S6 Table — (PDF) [file pone.0200683.s006.pdf]

S6 Table. Patients' disposition for Stage 2 (clinical assessment) of the HS Epidemiology study

Patients' disposition	Suspected HS (N = 88)
Did not consent to sharing their contact information	49/88 (55.7%)
Incorrect details provided	1/88 (1.1%)
Did not agree to attend dermatology consult	15/88 (17.0%)
Did not attend dermatology consult once appointment was scheduled. Attempts to reschedule were unsuccessful.	11/88 (12.5%)
Attended dermatology clinic as scheduled (evaluable)	12/88 (13.6%)
