# Supplementary material for: Hidradenitis Suppurativa (HS) prevalence, demographics and management pathways in Australia: A population-based cross-sectional study
Source: PLoS One. 2018 Jul 24;13(7):e0200683. doi: 10.1371/journal.pone.0200683 (PMC6057625; doi:10.1371/journal.pone.0200683)
Supplement: S7 Table — (PDF) [file pone.0200683.s007.pdf]

S7 Table. Demographic characteristics of suspected HS individuals [a]: Attended vs not attended clinic

		Attended (N=12)	Not attended (N=76)	Total (N=88)	P-value
Gender	Male	6/12 (50.0%)	24/76 (31.6%)	30/88 (34.1%)	0.2109
	Female	6/12 (50.0%)	52/76 (68.4%)	58/88 (65.9%)	
Age category	18-24 years	0	6/76 (7.9%)	6/88 (6.8%)	0.3812
	25-34 years	2/12 (16.7%)	13/76 (17.1%)	15/88 (17.0%)	
	35-44 years	4/12 (33.3%)	25/76 (32.9%)	29/88 (33.0%)	
	45-54 years	2/12 (16.7%)	17/76 (22.4%)	19/88 (21.6%)	
	55-64 years	4/12 (33.3%)	9/76 (11.8%)	13/88 (14.8%)	
	65 years & over	0	6/76 (7.9%)	6/88 (6.8%)	
Country of Birth	Australia	10/12 (83.3%)	66/76 (86.8%)	76/88 (86.4%)	0.7420
	Other	2/12 (16.7%)	10/76 (13.2%)	12/88 (13.6%)	
Household location	VIC	5/12 (41.7%)	21/76 (27.6%)	26/88 (29.5%)	0.2890
	NSW/ACT	5/12 (41.7%)	19/76 (25.0%)	24/88 (27.3%)	
	QLD	0	14/76 (18.4%)	14/88 (15.9%)	
	SA	0	11/76 (14.5%)	11/88 (12.5%)	
	WA	1/12 (8.3%)	8/76 (10.5%)	9/88 (10.2%)	
	NT/TAS	1/12 (8.3%)	3/76 (3.9%)	4/88 (4.5%)	
Smoker	No	7/12 (58.3%)	33/76 (43.4%)	40/88 (45.5%)	0.3350
	Yes	5/12 (41.7%)	43/76 (56.6%)	48/88 (54.5%)	
Highest education level	High-school	3/12 (25.0%)	40/76 (52.6%)	43/88 (48.9%)	0.0752
	University	9/12 (75.0%)	36/76 (47.4%)	45/88 (51.1%)	
Personal Annual Income	Less than 20K (AUD)	2/12 (16.7%)	19/76 (25.0%)	21/88 (23.9%)	0.0321
	20K-<40K (AUD)	3/12 (25.0%)	32/76 (42.1%)	35/88 (39.8%)	
	40K-<80K (AUD)	3/12 (25.0%)	20/76 (26.3%)	23/88 (26.1%)	
	80K(AUD) or more	4/12 (33.3%)	5/76 (6.6%)	9/88 (10.2%)	
Employment status	Employed	6/12 (50.0%)	34/76 (44.7%)	40/88 (45.5%)	0.4689
	Unemployed	4/12 (33.3%)	14/76 (18.4%)	18/88 (20.5%)	
	Retired	2/12 (16.7%)	13/76 (17.1%)	15/88 (17.0%)	
	Student	0	2/76 (2.6%)	2/88 (2.3%)	
	Home duties	0	13/76 (17.1%)	13/88 (14.8%)	
Occupation	Professional/Semi-pro/Sales	2/6 (33.3%)	9/34 (26.5%)	11/40 (27.5%)	0.3556
	Executive/White collar	4/6 (66.7%)	16/34 (47.1%)	20/40 (50.0%)	
	Skilled/Semi-skilled	0	9/34 (26.5%)	9/40 (22.5%)	
	Unskilled/Farm owner/worker	0	0	0	
	Unclassified	0	0	0	
Marital status	Married/De Facto	5/12 (41.7%)	41/76 (53.9%)	46/88 (52.3%)	0.7311
	Single/Separated/Engaged/ Planning to marry	5/12 (41.7%)	25/76 (32.9%)	30/88 (34.1%)	
	Widowed/Divorced	2/12 (16.7%)	10/76 (13.2%)	12/88 (13.6%)	
BMI category	Underweight	0	0	0	0.2695
	Acceptable weight	2/7 (28.6%)	3/23 (13.0%)	5/30 (16.7%)	
	Overweight	3/7 (42.9%)	4/23 (17.4%)	7/30 (23.3%)	
	Obese	2/7 (28.6%)	14/23 (60.9%)	16/30 (53.3%)	
	Unclassified	0	2/23 (8.7%)	2/30 (6.7%)	

a Individuals suspected of having HS (N=88) based on the results of the HS screening questionnaire (Table S1).
